# Maxillary Melanotic Neuroectodermal Tumor of Infancy

**DOI:** 10.5005/jp-journals-10005-1021

**Published:** 2009-12-26

**Authors:** Marcio A da Fonseca, S Thikkurissy

**Affiliations:** 1 Clinical Professor, Division of Pediatric Dentistry, Ohio State University/Nationwide Children’s Hospital, Columbus, OH; 2 Assistant Professor, Division of Pediatric Dentistry, Ohio State University/Nationwide Children’s Hospital, Columbus, OH

**Keywords:** Melanotic neuroectodermal tumor of infancy, maxilla, pediatric dentistry, pediatric oral pathology.

## Abstract

The melanotic neuroectodermal tumor of infancy (MNTI) is a rare benign neoplasm of neural crest origin most commonly found in the anterior region of the maxilla. The tumor almost always develops during the first year of life, although in some cases it can be present at birth. MNTI’s present as a rapidly growing, painless expansile, partly pigmented mass. They are usually unencapsulated, with a tendency to occur as a single lesion. Local excision is the treatment of choice and is usually curative. Main sites for recurrences are the maxilla (57%) and the skull/brain (28.6%). Malignant transformation has been noted in approximately 6.5% of all cases and in 2% of maxillary tumors. The case of a 3-month old boy who presented with a loose primary maxillary left central incisor is discussed. The diagnostic and clinico-pathological features as well as tumor management and importance of a timely diagnosis are reviewed.

## INTRODUCTION

First described by Krompecher in 1918, the melanotic neuroectodermal tumor of infancy (MNTI) is a rare benign neoplasm of neural crest origin.^1^,^2^ It is most commonly found in the anterior region of the maxilla but has also been described in the mandible, brain, skull, epididymis, shoulder, scapula, fontanel, and mediastinum^3^,^4^ MNTI almost always develops during the first year of life, usually in the first 6 months, although in some cases it can be present at birth.^4^,^5^ The classic presentation is of a rapidly expanding mass that may have a blue, brown or black coloration.^5^ They present osteolytic lesions with associated dissolution and degeneration of surrounding bony tissues, often associated with displacement of developing teeth.^5^

The case of a 3 months old boy who presented with a complaint of a loose primary maxillary left central incisor is discussed. The diagnostic and clinico-pathological features as well as tumor management and importance of a timely diagnosis are reviewed.

## CASE REPORT

A 3-month old caucasian boy was referred to the dental clinic at a major children’s hospital by a physician in the emergency department (ED). His young parents had several concerns: (1) "Swollen gums" in the upper anterior area; (2) A loose upper left incisor (#F) that had erupted quickly in just a few weeks; (3) Feeding disturbances caused by the tooth; (4) Fear that it might be aspirated.

The boy was healthy and had no known drug allergies. He was afebrile, playful and active. The extraoral exam was not remarkable. The intraoral exam revealed the primary maxillary left central incisor erupted, very mobile (grade 3), with edematous gingiva surrounding it. No other teeth or swelling were present although the incisal edge of the primary mandibular right central incisor could be felt on palpation. His parents wanted the tooth removed and declined a radiograph. After a written consent was obtained, a pediatric dental resident infiltrated 0.5 ml 2% lidocaine with epinephrine 1:100,000 around the primary maxillary left central incisor which was extracted uneventfully via forceps. Hemostasis was easily obtained with a resorbable gelatin sponge and gauze pressure. Postoperative instructions were reviewed with the parents and all questions were answered. The tooth presented a fully developed crown but no root structure.

**Fig. 1:**
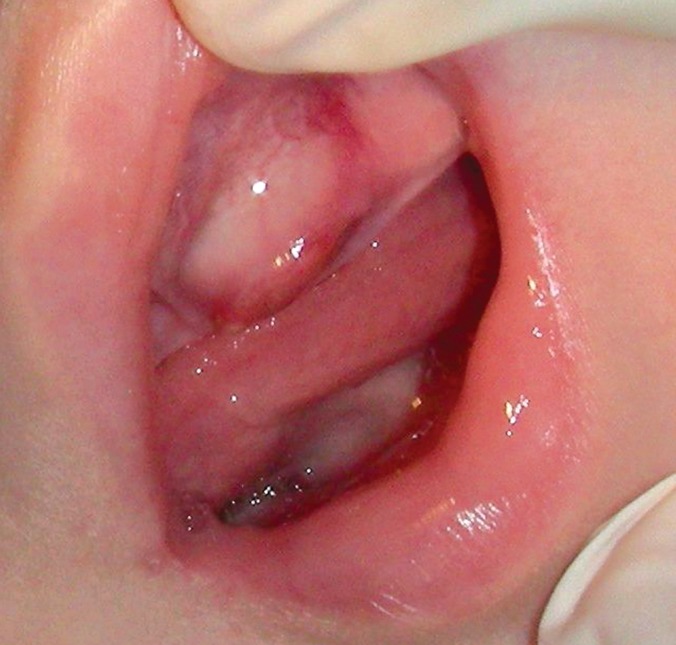
The extraoral examination showing left facial assymmetry with nostril and upper lip being displaced upwards with evident bulging of the area

**Fig. 2:**
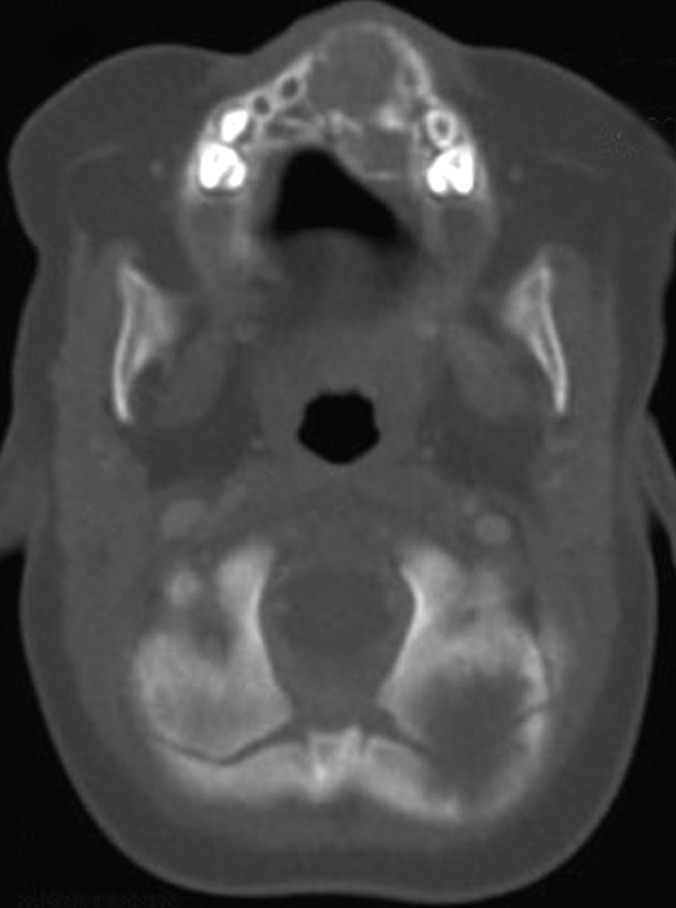
The nosal examination revealing expansion of the upper left arch, involving both vestibular and palatal cortices

**Fig. 3:**
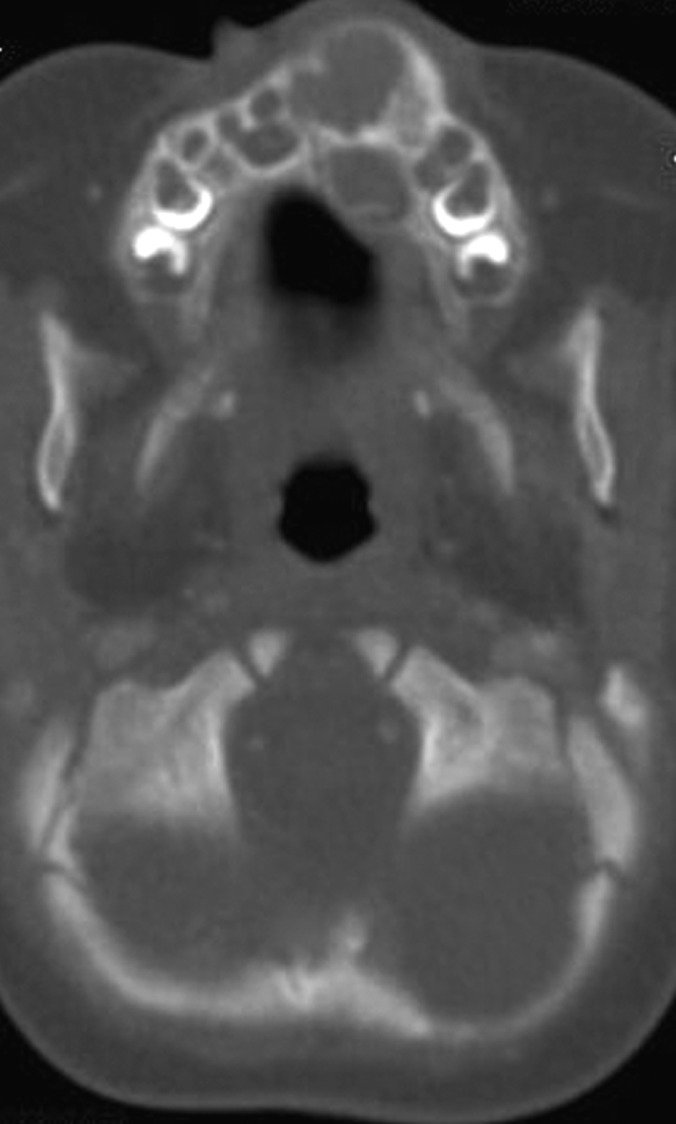
CT scan of the facial bones

Eleven days later, the pediatric dental resident on call was summoned to the ED at 11 pm. The parents had brought the patient back with complaints of gingival and nasal bleeding, and facial swelling which increased in size steadily after the extraction. The patient was not feeding well, had lost weight and was dehydrated. The extraoral exam showed left facial asymmetry with the nostril and upper lip being displaced upward with evident bulging of the area (Fig. 1). The intraoral exam revealed expansion of the upper left arch, involving both the vestibular and palatal cortices, extending from the anterior region to the first primary molar area, filling the upper gingivobuccal sulcus (Fig. 2). The area was firm, nontender, blue/black in color with purulent exudate present in the extraction site. A computerized tomography (CT) scan of the facial bones revealed an expansile, bilobed mass with soft tissue attenuation centered along the left upper anterior area, measuring 14 mm mediolaterally at the region of the incisors and 8 mm mediolaterally in the hard palate (Fig. 3). Cortical thinning and destruction of the maxilla and hard palate were evident. The orbits were intact and there was no sign of lymphadenopathy.

The patient was admitted to the hospital and the oral and maxillofacial surgery and oral pathology services were contacted for a consult. A radical excisional biopsy was performed under general anesthesia by the oral surgeon the following day and two specimens were sent to the lab for examination. The buds of the primary maxillary left lateral incisor and canine as well as the permanent maxillary left central incisor, lateral incisor and canine were removed with the mass. The sections studied showed a large number of nests with lesional cells within a fibrous stroma. Two types of cells were described: (1) A large cell population that was highlighted by cytokeratin and immunostains which was consistent with neural, epithelial and melanotic markers; (2) A population of small, round blue cells that were "very neuroblastic" in appearance. The final diagnosis was MNTI.

Following the surgery, the facial asymmetry disappeared and the boy was able to feed well. The postoperative course was uneventful, and he was scheduled to follow-up with the oral surgeon 2 weeks later and at regular intervals throughout the first year to monitor for recurrences. At discharge, the pediatric dental service reviewed oral hygiene, diet and caries prevention with the parents as well as future orofacial rehabilitation the patient may need. He will return to the pediatric dental clinic at 1 year of age to establish a dental home and will then be referred to the hospital craniofacial team for follow-up on his craniofacial growth.

## DISCUSSION

Early diagnosis and treatment of any pathological condition is the key to avoid serious sequelae. Late diagnosis of tumors with rapid invasive growth, such as in the case of TMNI, may compromise a radical resection because of the possible involvement of the adjacent critical anatomic structures. Although our patient did not present a clear bony expansion at the time of the extraction, a high index of suspicion would have led to a quicker identification and resolution of the problem. Firstly, the gingival tissue surrounding the primary maxillary left central incisor did not have a healthy presentation. Secondly, the initial thought of the erupted tooth being a neonatal one was erroneous given that those teeth erupt within 30 days of birth. In this case, the patient’s tooth erupted around 80-90 days of age. Furthermore, the fully developed crown should have led to the suspicion of a normal primary tooth being displaced and forced to an early eruption by a pathological entity. Neonatal teeth rarely present fully developed crowns. A radiograph of the upper anterior area would have shown the lytic lesion and tooth displacement which would have generated an immediate consult with the other dental services before the dental extraction.

MNTI’s present as a painless, expansile, partly pigmented mass. They are usually unencapsulated and tend to occur as a single lesion.^6^ They are found most commonly in the maxilla (61.4%) and occasionally in the mandible (6.4%).^6^ Ninety percent of them are diagnosed prior to one year of age, with a mean of 4.3 months.^6^-^8^ Some cases have been reported in older children and adults but the oral cavity and the jaws were not involved in these patients.^6^,^8^ Most reports show no gender predilection; however, a review of 140 cases revealed a male/female ratio of 1.48.^6^ Although MNTI’s are mostly benign neoplasms of early infancy, they present a malignant potential given their (1) Fairly aggressive expansion which may obstruct the infant airway, (2) High recurrence rate of 20 to 50%, and (3) 5-10% metastatic rates.^6^,^8^ Main sites for recurrences are the maxilla (57%) and the skull/brain (28.6%).^6^ Relapses in the jaws are seen within the first 6 months of life while the peak of recurrences in the skull and brain is found in older patients.^6^ In the extensive series reviewed by Kruse-Losler et al,^6^ most of them happened within the first or second month after the surgical intervention. Recurrences may be due to the challenge of finding the right balance between radical surgical resection and preservation of important anatomical structures. MNTI’s diagnosed at a very early age (in the first few days of life) may present a more aggressive behavior.^9^ Malignant transformation has been noted in approximately 6.5% of all cases and in 2% of maxillary tumors.^3^,^5^,^6^

Since its first description, MNTI has been assigned more than 20 other names, such as pigmented ameloblastoma, retinal anlage tumor and melanotic progonoma, because of the variable theories of its origin. The current nomenclature was arrived at in 1966 when Borello and Gorlin^10^ noted high urinary concentrations of vanillyl mandelic acid (VMA) in patients diagnosed with the tumor. The increased excretion of VMA, which is a major urinary metabolite, is an indicator for neural crest tumors such as pheochromocytomas and neuroblastomas.^10^,^11^ VMA values may return to normal once the tumor has been resected.^5^ Interestingly, while the tumors Borello and Gorlin studied exhibited this property, other cases of MNTI have failed to produce significant levels of VMA. Our patient was not tested for it.

Radiographic examination of MNTI’s usually reveals a radiolucency with or without regular margins.^6^ In some cases, an associated osteogenic reaction, which exhibits a "sun ray" radiographic pattern, may be seen and may lead to a misdiagnosis of osteosarcoma.^5^ CT scanning tends to show hyperdense masses and can accurately define the extent of the lesion, showing tooth displacement, as seen in this case.^6^,^12^ Magnetic resonance imaging with gadolinium contrast provides optimal tissue characterization while avoiding the radiation exposure of a CT scan.^12^ The ectodermal band that ultimately gives rise to the dental lamina is intimately associated with neuroectodermal cells which are theorized to be the origin of the MNTI.

While clinical and radiographic findings may be suggestive of the tumor, histopathological examination is required for a definitive diagnosis. MNTI consists of 2 different cell populations that form nests, tubules and alveolar structures within a dense, collagenous stroma. These structures are lined by cuboidal epithelial cells that have granules of dark brown melanin pigment. The other cell type is neuroblastic in appearance, consisting of small round cells.^5^ The differential diagnosis for MNTI include, among others, benign fibromatosis, giant cell granuloma, and pigmented intraosseous odontogenic carcinoma of the maxilla. Aggressive, small round cell tumors such as neuroblastoma, lymphomas, Ewing’s sarcoma and rhabdomyosarcoma must be also taken into account. The biphasic cell distribution described above distinguishes the MNTI from these other neoplasms.

Local excision is the treatment of choice and is usually curative.^12^ Some clinicians prefer simple curettage, although others advocate that a 5 mm margin of normal tissue should be obtained.^5^ Excision involves largely blunt dissection of the egg-shell thin fibro-osseous capsule from the thinned and expanded hard palate.^12^ Recurrences require re-excison.^12^ In cases where absolute surgical eradication is not possible, such as in midline lesions or those approximating the cranial base, chemotherapy and radiotherapy are potential alternatives.^6^,^13^ However, this is controversial. Shaia et al^9^ stated that the utility of these types of therapy for MNTI management is unclear and that they should be reserved for metastatic disease, cases of recurrence after a second resection, or when complete excision is not possible. Others say that no role of radiation therapy or chemotherapy has been reported for initial or recurrent cases of palatal MNTI.^12^ Dental and palatal reconstruction should be reconsidered at approximately 6 years of age, when the permanent dentition is erupting, to ensure an optimal periodontal uptake.^12^

In summary, the case of a 3-month old male with MNTI associated with premature eruption of a primary tooth is presented. Despite a short delay in diagnosing the entity, its complete surgical removal without involvement of vital structures was possible. The patient will be followed by the hospital’s transdisciplinary craniofacial team to treat resulting eruption aberrances and to monitor for recurrences, given the potential serious nature of this entity.
